# Transcriptomic Analyses Reveal Novel Genes with Sexually Dimorphic Expression in Yellow Catfish (*Pelteobagrus fulvidraco*) Brain

**DOI:** 10.1007/s10126-015-9650-z

**Published:** 2015-08-05

**Authors:** Jianguo Lu, Min Zheng, Jiajia Zheng, Jian Liu, Yongzhuang Liu, Lina Peng, Pingping Wang, Xiaofeng Zhang, Qiushi Wang, Peixian Luan, Shahid Mahbooband, Xiaowen Sun

**Affiliations:** Heilongjiang River Fisheries Research Institute, Chinese Academy of Fishery Sciences, 43 Songfa Street, Daoli District, Harbin, 150070 China; School of Computer Science and Technology, Harbin Institute of Technology, Harbin, China; Department of Civil Engineering, Auburn University, Auburn, AL 36849 USA; National and Local United Engineering Lab for Freshwater Fish Breeding, Harbin, China; Harbin Normal University, Harbin, China; Department of Zoology, College of Science, King Saud University, Riyadh, 11451 Saudi Arabia

**Keywords:** Transcriptome, Sexually dimorphic, Yellow catfish, RNA-Seq

## Abstract

Yellow catfish (*Pelteobagrus fulvidraco*) is a pivotal freshwater aquaculture species in China. It shows sexual size dimorphism favoring male in growth. Whole transcriptome approach is required to get the overview of genetic toolkit for understanding the sex determination mechanism aiming at devising its monosex production. Beside gonads, the brain is also considered as a major organ for vertebrate reproduction. Transcriptomic analyses on the brain and of different developmental stages will provide the dynamic view necessary for better understanding its sex determination. In this regard, we have performed a de novo assembly of yellow catfish brain transcriptome by high throughput Illumina sequencing. A total number of 154,507 contigs were obtained with the lengths ranging from 201 to 27,822 bp and N50 of 2,101 bp, as well as 20,699 unigenes were identified. Of these unigenes, 13 and 54 unigenes were detected to be XY-specifically expressed genes (SEGs) for one and 2-year-old yellow catfish, while the corresponding numbers of XX-SEGs for those two stages were 19 and 13, respectively. Our work identifies a set of annotated genes that are candidate factors affecting sexual dimorphism as well as simple sequence repeat (SSR) and single nucleotide variation (SNV) in yellow catfish. To validate the expression patterns of the sex-related genes, we performed quantitative real-time PCR (qRT-PCR) indicating the reliability and accuracy of our analysis. The results in our study may enhance our understanding of yellow catfish sex determination and potentially help to improve the production of all-male yellow catfish for aquaculture.

**Electronic supplementary material:**

The online version of this article (doi:10.1007/s10126-015-9650-z) contains supplementary material, which is available to authorized users.

## Introduction

Yellow catfish (*Pelteobagrus fulvidraco*) is an important freshwater aquaculture species in China. Genetically, yellow catfish harbors an XX/XY system (Liu et al. 2010, 2013). It displays sexual size dimorphism, male exhibits faster growth rate and reaching a larger ultimate size (three times) than female (Park et al. 2004; Liu et al. 2013). Apart from the intriguing reproductive biology, it is economically vital with continuously growing aquaculture in China but limited available genetic resources. Efficient utilization of the natural diversity of trait phenotypes, i.e., utilization of growth trait to produce monosex of yellow catfish, requires the identification of genetic underpinnings of trait differences. To get an overview of the genetic toolkit deployed for the development and maintenance of the differences between sexes, whole transcriptome approach is required (Piferrer et al. 2012).

In our previous study (Lu et al. 2014), we have identified sex-related genes from gonad transcriptomic analysis which has allowed us to profile the expression of a series of genes involved in sex differentiation of female and male yellow catfish. However, sex determination gene and sex determination mechanism have yet to be elucidated. Similar analyses on other tissue parts and of different developmental stages will provide the dynamic view necessary for a better understanding of sex determination.

To our knowledge, beside gonads, the brain is also involved in vertebrate reproduction. It controls growth and reproduction mainly through the brain–pituitary–gonadal axis (Weltzien et al. 2004). The involvement of the brain in gonad development has been established by studies on quail (Munday et al. 2006; Francis and Barlow 1993), indicating the brain may determine the fate of the gonads (Sreenivasan et al. 2008). It is also reported that differential brain gene expression can also lead to different development of the brain in the two sexes; this even occurs before the gonads formed (Dennis 2004; Dewing et al. 2003; Davies and Wilkinson 2006; Sellars et al. 2015). Also, the brain exhibits one of the most complex transcriptomes of all organs in vertebrates; hence, it is a tissue of choice for sequencing a maximum number of transcripts while reducing the need for normalization (Tzika et al. 2011).

So far, many sex-related genes in brain have been detected. In rodents, Sry was a male-determining gene expressed in the substantia nigra of the midbrain (Dewing et al. 2006). In zebra finch, the expression level of trkB in male brain, which was a Z-linked gene, is found to be higher than in female (Chen et al. 2005). More genes have been identified showing sex-related expression pattern in the brain including Usp9x/y (Xu et al. 2002), Utx/Uty (Xu et al. 2008), CHD1Z/W (Agate et al. 2003, 2004), and sex-specific markers (Xu et al. 2013), whereas their roles in sex determination are yet not known. Not only in rodents and birds, sex-related genes in brain were also found in teleost fish species, including medaka (Maehiro et al. 2014), zebrafish (Sreenivasan et al. 2008; Santos et al. 2008), rainbow trout (Vizziano-Cantonnet et al. 2011; Yano et al. 2014), sharpsnout seabream (Manousaki et al. 2014), and goldfish (Parhar et al. 2001).

To gain further insight into molecular mechanism underlying yellow catfish’s sexual dimorphism, we extended our studies by assembling brain transcriptome of yellow catfish using in-depth Illumina HiSeq sequencing.

In our study, we sequenced yellow catfish brain transcriptome of two developmental stages and identified the differences in gene expression profiles between sexes. More novel genes were identified related to sex dimorphism in the brain as well as genetic markers. The further identification of the expression profile of genes involved in sex dimorphism may help to illuminate the gene regulatory network controlling sex determination and subsequent maintenance of phenotypic sex and to devise the production of monosex yellow catfish (Liu et al. 2013; Chen et al. 2015).

## Materials and Methods

### Ethics Statement, Experimental Fish, and Sample Collection

All procedures in our study including the handling and treatment of fish were approved by the Animal Care and Use Committee at Heilongjiang River Fisheries Research Institute (ACUC-HRFRI). We used 40 fishes, including 20 male yellow catfish (10 one-year-old juveniles, 10 two-year-old adults) and 20 female yellow catfish (10 one-year-old juveniles and 10 two-year-old adults). They are full-sibling yellow catfish which came from the National Fish Original Species Farm at Zhaodong City, Heilongjiang Province, China. One-year-old and 2-year-old yellow catfish were collected in August 16, 2012 and August 14, 2013, respectively. The sex of these fish was confirmed anatomically. Then, the experimental fish were euthanized with 250 mg/L tricaine methanesulfonate before sample collection. Brain tissues of male and female yellow catfish from two developmental stages were collected and stored in 1.5 ml RNAlater tube (Qiagen, Hilden, Germany), respectively, and then transferred to a −80 °C freezer until prior to RNA extraction.

### RNA Extraction

Samples were removed from the −80 °C freezer and homogenated using TissueRuptor (Qiagen, Hilden, Germany) to a fine solution. Total RNAs were extracted from each sample using RNeasy Lipid Tissue Kit (Qiagen, Hilden, Germany) according to the manufacturer’s instructions after the RNase-free DNase I (Qiagen, Hilden, Germany) treatment of genomic DNA elimination. The concentration and integrity of RNA were examined using Thermo Scientific™ NanoDrop™ 8000 Spectrophotometer and Agilent 2100 Bioanalyzer (Agilent Technologies, Santa Clara, CA, USA). The RNA with OD260/280 ≥1.8 and RNA integrity number (RIN) ≥7.0 was selected for the following experiment. Equal amounts of the high-quality RNA samples from brain tissue were then pooled together for cDNA synthesis and sequencing.

### Library Construction, Illumina Sequencing, and Assembly of cDNA Library

RNA-Seq library preparation and sequencing were carried out by BerryGenomics sequencing company (Beijing, China). cDNA library was prepared with ~2.5 μg of total RNA based on the Illumina TrueSeq RNA Sample Preparation Kit (Illumina) protocols. The library was then amplified, and the final library yields ~400 ng with average fragment size of ~270 bp. The library was sequenced with one lane on an Illumina HiSeq 2000 instrument with 100-bp paired-end reads after KAPA quantitation and dilution. Raw read data of yellow catfish RNA-Seq with SRA format have been uploaded to the NCBI Short Read Archive under the accession number SRR1103702. The clean reads from the four transcriptome were filtered out adaptor-only reads and low-quality reads (reads with *Q* value ≤20). Cleaned reads were used for de novo assembly using the de Brujin graph approach with Trinity by default parameters.

### Transcriptome Construction and Assembly Assessment

To assess the transcriptome of yellow catfish, we compared the transcriptome contigs with Refseq and Ensemble proteins of zebrafish, medaka, stickleback, and tetraodon by BLAST program with default parameters.

### Functional Annotation and Gene Ontology Analysis

The assembly RNA-Seq contigs was used for similarity search program against reference protein sequence including zebrafish, medaka, stickleback, and tetraodon, respectively. The similarity searches were performed using the BLASTX program with the *E* value cutoff of 1e-10. Gene annotation was assigned to the RNA-Seq contigs based on the top BLAST hit. Gene ontology (GO) annotation and enrichment analysis was then followed using Blast2GO. The level 2 GO terms associated with transcriptome contigs were retrieved, and then, the annotation result was categorized as biological process, molecular function, and cellular components.

### Gene Expression and Differentially Expressed Gene

Audic’s method was used to identify differentially expressed genes between two libraries (Audic and Claverie 1997). The threshold for the *P* value was determined using false discovery rate (FDR) and was widely set at 0.05 (Benjamini and Yekutieli 2001). In this study, “FDR < 0.05” and “XX_FPKM = 0 or XY_FPKM = 0” were used to classify as specifically expressed genes (SEGs). “FDR < 0.05” and “|log_2_(XX_FPKM/XY_FPKM)| > 1 or |log_2_(XY_FPKM/XX_FPKM)| > 1” were used to classify as differentially expressed genes (DEGs). Those meeting “XX_FPKM < 2 and XY_FPKM < 2” statistical criteria were classified as non-expressed genes (NEGs), whereas all there remaining ones were designated as co-expressed genes (CEGs). In this way, all genes were classified into four types: SEGs, DEGs, CEGs, and NEGs.

### SSR and SNV Detection

The SSR detection was applied using msatcommander toolkits (Faircloth 2008). The sequences composed of two or more repeat units with motifs separated by 100 bp were considered to be two or more microsatellites. Only microsatellite sequences with flanking sequences of >50 bp on both sides were collected for future primer designing. The SNP and structure variation detection was employed using bwa/SAMtool (Li et al. 2009).

### Experimental Validation Using qRT-PCR

To verify the expression profile of the differentially expressed genes selected using bioinformatics method, we have performed quantitative real-time PCR (qRT-PCR). Total RNAs were reverse-transcribed using reverse transcriptase (Invitrogen, Carlsbad, CA, USA). All primers were designed using Primer Premier 6. The SYBR Green I Master Mix (TaKaRa, Dalian, China) was used for qRT-PCR using an Applied Biosystems Prism 7500 Fast Real-Time PCR system. One denaturation cycle was performed at 95 °C for 5 min, and 40 amplification cycles were performed at 95 °C for 15 s and 60 °C for 30 s. Results were expressed relative to the expression levels of beta-actin in each sample using the Relative Expression Software Tool (REST) version 2009. Expression differences between groups were assessed for statistical significance using R software. All samples were run in triplicate. The averages of three relative quantities of biological replications were used in a two-tailed Student’s *t* test with a 95 % confidence level (*P* < 0.05) to determine the significance with respect to gene expression.

## Results

### Transcriptome Sequencing and Assembly

**Fig. 1:**
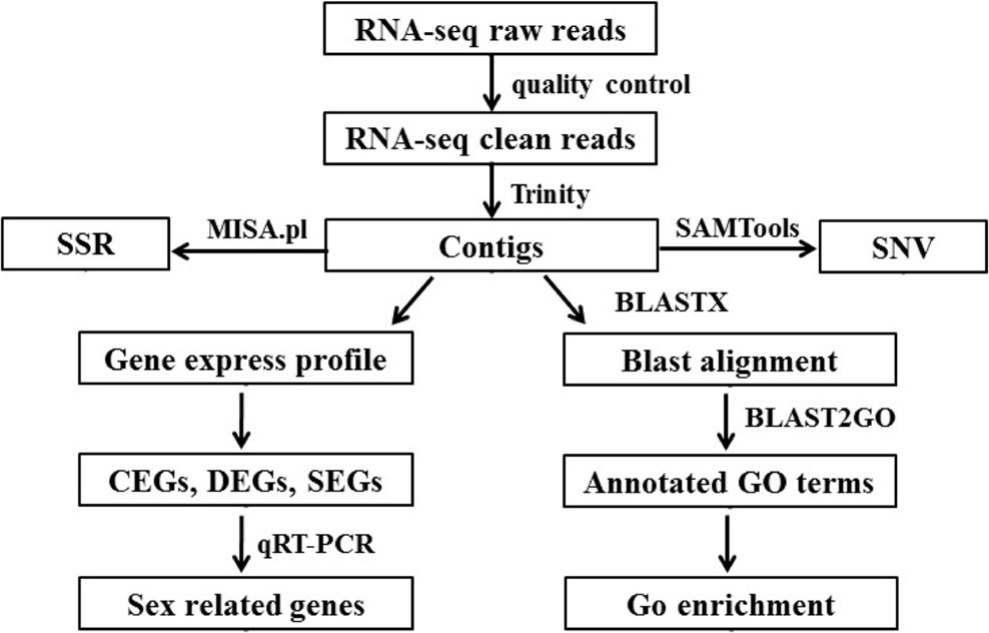
Yellow catfish transcriptome analysis pipeline

To generate a comprehensive reference transcriptome of the yellow catfish and to investigate gene expression variations between sexes in the brain, we prepared four cDNA libraries from male and female pooled brain RNAs of 1-year- and 2-year-old yellow catfish, and then they were sequenced by using Illumina HiSeq 2000 technology. In total, 209,599,618, 100-bp-long paired-end reads were obtained. Then, the low-quality sequences and ambiguous nucleotides were removed, with remaining 191,071,480 clean reads for Trinity de novo assembling (Table 1). A total of 154,507 contigs were assembled, ranging from 201 to 27,822 bp with the average length of 1,014.05 bp, the N50 value of 2,101 bp, and the N90 value of 349 bp (Table 1). The raw transcriptome sequences in this study have been uploaded in the NCBI SRA site with the accession number SRR1103702. In addition, the percentage of GC content was 46.58 %. The transcriptome data analysis workflow was shown in Fig. 1.

**Table 1:** Statistical summary of yellow catfish transcriptome data

Sequencing	Number of reads	209,599,618
	Total bases	20.96 Gb
	Cleaned reads	191,071,480
Assembly	Number of contigs	154,507
	Average contig length	1,014.05
	N50 value	2,101
	N90 value	349
Annotation	Contigs with blast results	150,291
	Unigenes with blast result	27,226
	Contig with GO terms	64,794
	Unigenes with GO terms	19,039

### Gene Identification and Functional Annotation

BLAST-based gene identification was performed to annotate the yellow catfish transcriptome and identify genes with sexually dimorphic expression. BLASTX was used for the assembled contig gene identification by searching against the reference protein sequences, including zebrafish (*Danio rerio*), medaka (*Oryzias latipes*), stickleback (*Gasterosteus aculeatus*), and tetraodon (*Tetraodon nigroviridis*). There were 150,291 contigs corresponding to 27,226 unigenes, with an *E* value cutoff less than 1e-10. A total number of 64,794 assembled contigs were assigned at least one GO term, corresponding to 19,039 unigenes for describing biological processes, molecular functions, and cellular components (Table 1).

**Fig. 2:**
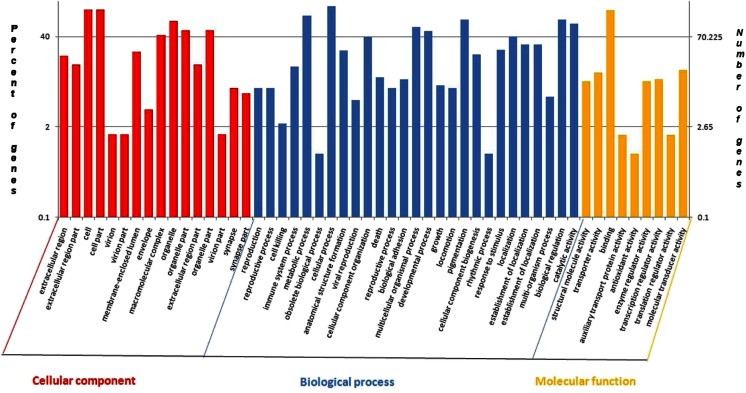
Gene ontology categories pattern of the differentially expressed unigenes in yellow catfish. Distribution of the GO categories assigned to the differentially expressed unigenes in yellow catfish. Unique transcripts (unigenes) were annotated in three categories: cellular components, molecular functions, and biological processes

The differentially expressed unique genes were then used as input to perform GO annotation by Blast2GO (Conesa et al. 2005). The GO annotations were plotted in Fig. 2. Of these, the number of GO terms in biological process ontology was the most prevalent, followed by the cellular component ontology and the molecular function. Briefly, for biological processes, genes involved in cellular processes and metabolic process were highly represented. For the cellular component, cell and cell part were the most represented categories. Binding was the most represented GO term, followed by molecular transducer activity for molecular functions. Interestingly, within biological processes, a total of 11 unigenes were annotated to reproduction (GO, 0000003), 88 unigenes were annotated to developmental process (GO, 0032502), 12 unigenes were annotated to growth (GO, 0040007), and 11 unigenes were annotated to reproductive process (GO, 0022414).

Then, we performed sex-biased GO term analysis. For male-biased genes, the significant enrichment GO terms were related to nucleoside/ribonucleoside and other complex catabolic process, responding to environment or ion. Meanwhile, the female-biased GO terms were related to DNA modification, meiosis, and cell adhesion. Interestingly, many female-biased GO terms were annotated to female gamete generation (GO, 0007292), oogenesis (GO, 0048477), and germplasm (GO, 0060293). The sex-biased GO terms were shown in Supplementary Tables 1 and 2.

### Assessment of Transcriptome Assembly

In order to assess the quality of the yellow catfish transcriptome assembly, the assembled contigs were compared with Refseq proteins of zebrafish, medaka, stickleback, and tetraodon using BLASTX with an *E* value cutoff of 1e-10. In total, 80,659 (52.2 %), 66,344 (42.9 %), 68,871 (44.6 %), and 65,507 (42.4 %) significant hits were identified with those fish species, respectively (Table 2). In addition, the total numbers of 16,351, 10,989, 12,508, and 11,523 unigenes were retained for zebrafish, medaka, stickleback, and tetraodon after filtering the repeat isogenes. The corresponding uniprotein numbers of these four fish are 20,978 (zebrafish), 15,935 (medaka), 16,918 (stickleback), and 15,579 (tetraodon), respectively (Table 2). We again noted that zebrafish had the highest similarity to yellow catfish, which is consistent with our previous findings (Lu et al. 2014).

**Table 2:** The assessment of yellow catfish transcriptome assembly

Fish species	Blast hits number	Unigene number	Uniprotein number
Zebrafish	80,659	16,351	20,978
Medaka	66,344	10,989	15,935
Stickleback	68,871	12,508	16,918
Tetraodon	65,507	11,523	15,579

Unigene number is the number of unique gene after removing duplicated genes. Uniprotein number is the number of unique protein after removing duplicated proteins

### SSRs and SNV Detection Through RNA Sequencing

For further assessment of the assembly quality and development of new molecular markers, including di-, tri-, tetra-, and penta-nucleotide SSRs with a minimum of four repetitions for all motifs, the SSRs that were only located in one single read had been filtered. A total number of 90,210 microsatellites were identified from 154,507 contigs. Among these microsatellites, di-nucleotide repeat motifs were the most abundant type (72,143, 80.0 %), followed by tri-, tetra-, and penta-nucleotide repeat motifs (13,676 (15.2 %), 4,273 (4.7 %), and 118 (1.3 %); see Table 3). Eight thousand seven hundred forty-five SSRs are gene associated. These SSRs provide plenty molecular information to design polymorphic primers for further genotyping analysis.

**Table 3:** Statistics of microsatellite identified from yellow catfish transcriptome

Total number of contigs	154,507
Di-nucleotide repeats	72,143
Tri-nucleotide repeats	13,676
Tetra-nucleotide repeats	4,273
Penta-nucleotide repeats	118
Microsatellite total number	90,210

For extended application of the RNA-Seq data, structure variations were discovered using the assembled transcriptome. The short reads of RNA-Seq data were aligned onto the reference transcriptome of yellow catfish, and the total number of insertion and deletion (INDEL) varies from 2,343 to 2,992 from four different yellow catfish samples. The insertion number varies from 1,661 to 2,043, and the deletion number varies from 682 to 949, respectively. The total number of SNPs varies from 16,440 to 23,641 from four yellow catfish samples. The synonymous number varies from 2,493 to 4,604, and the non-synonymous number varies from 11,836 to 21,148, respectively (Table 4). This availability of microsatellites and structure variants developed in this study serves as precious molecular markers for yellow catfish genetics research.

**Table 4:** Statistics of INDELs and SNPs identified from yellow catfish transcriptome

Sample	INDEL number			SNP number		
	Insertion	Deletion	Subtotal	Synonymous	Non-synonymous	Subtotal
YC1XX^a^	1,661	682	2,343	2,603	18,608	21,211
YC1XY^b^	2,043	949	2,992	2,493	21,148	23,641
YC2XX^c^	1,836	744	2,580	4,604	11,836	16,440
YC2XY^d^	1,877	749	2,626	4,586	12,546	17,132

^a^One-year-old female yellow catfish

^b^One-year-old male yellow catfish

^c^Two-year-old female yellow catfish

^d^Two-year-old male yellow catfish

### Sex-Related Gene Expression Profiling in Brain

**Fig. 3:**
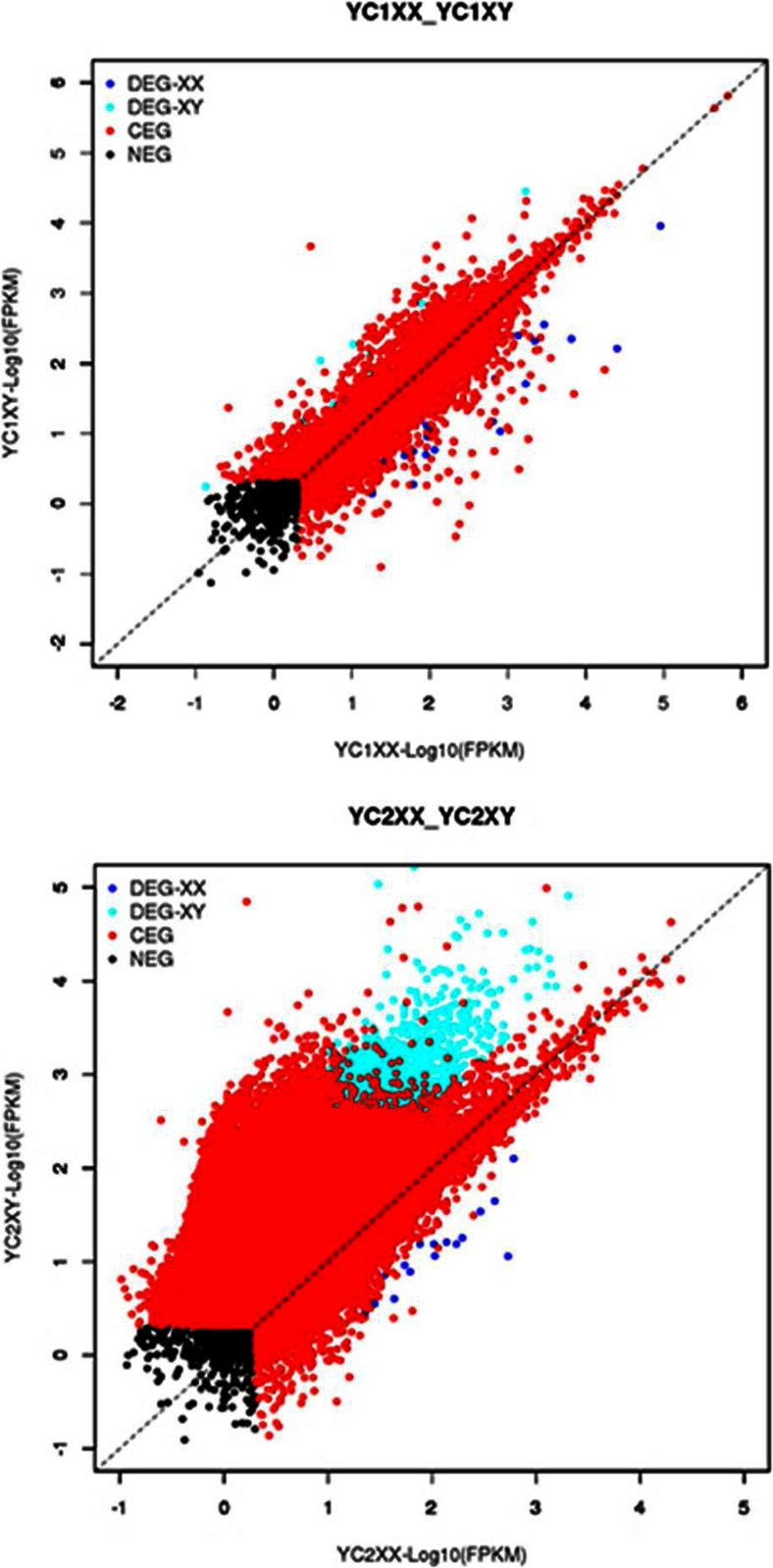
Gene expression profiling in gonad tissue at two developmental stages. The number of DEGs from two developmental stages was greater than SEGs. The number of DEGs was negatively correlated with that of CEGs expressed at two stages. More SEGs and DEGs were observed in XY brain in 2-year-old yellow catfish. However, in 1-year juvenile, more SEGs were detected in the female

According to the classification standard described in the method, a total of 27,226 unigenes were classified into four gene expression categories. For 1-year-old yellow catfish, 12,510 unigenes were identified as CEGs in both XX and XY brain transcriptome, and 39 and 19 unigenes were detected to be expressed differentially in XX or XY brain noted as DEGs, respectively (Table 5 and Fig. 3). For 2-year-old adults, 12,503 unigenes were detected as CEGs, and 113 and 186 unigenes were identified as DEGs in XX and XY, respectively. While the numbers of XX-SEGs at 1- and 2-year-olds were 19 and 13, respectively, the corresponding numbers for XY-SEGs were 13 and 54, respectively (Table 5 and Fig. 3).

**Table 5:** Three types of gene expression profile of yellow catfish

	CEGs	DEGs	SEGs
YC1XX	12,510	39	19
YC1XY		19	13
YC2XX	12,503	113	13
YC2XY		186	54

*CEGs* co-expressed genes, *DEGs* differentially expressed genes, *SEGs* specifically expressed genes

**Fig. 4:**
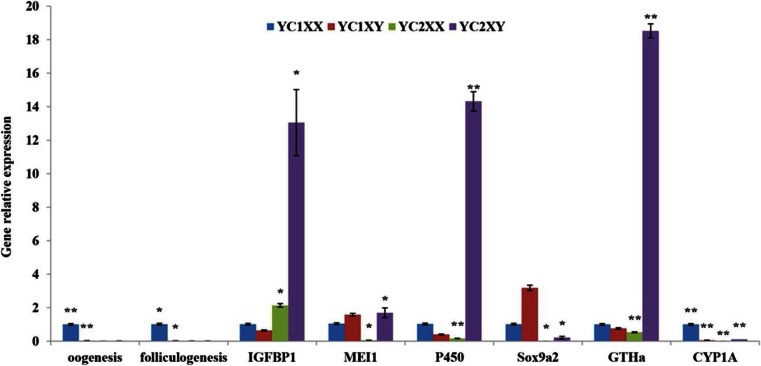
Validation of gonad tissue transcriptome results by qRT-PCR using eight selected differentially expressed genes in two developmental stages. qRT-PCR fold changes are relative to control samples and normalized by changes in beta-actin values. The averages of three relative quantities of biological replications were used in a two-tailed Student’s *t* test with a 95 % confidence level (*P* < 0.05) to determine the gene expression significance

The number of DEGs from two developmental stages was greater than SEGs. The number of DEGs was negatively correlated with that of CEGs expressed at two stages. More SEGs and DEGs were observed in XY brain in 2-year-old yellow catfish. However, in 1-year juvenile, more SEGs were detected in the female. The scatterplots of the gene expression profiles also revealed that there were more up-regulated genes in XY than in XX in 2-year-old but similar gene expression pattern of male and female in 1-year-old (Table 3 and Fig. 4).

Despite the significance in gene expression observed between sexes, CEGs at two developmental stages still made up the majority of all unigenes. However, compared to gonad (Lu et al. 2014), less DEGs and SEGs but more CEGs were found in the brain of yellow catfish.

### Identification of Sexually Dimorphic Expression Genes

The brain has pronounced sexual dimorphism in function in mammals. In 1-year-old brain tissue, four sex differentially overexpressed genes were detected in female, including CYP1A (Negrato et al. 2013; Hasselberg et al. 2004), ZP3 (Yuan et al. 2013; Mold et al. 2009), org (oogenesis-related gene), and lyceraldehyde 3-phosphate dehydrogenase (GAPDH). Sperm acrosome-associated 4 genes (SPACA4) (Shetty et al. 2003) as the only DEG was identified to be overexpressed in male (Table 6). The FIGLA gene was specifically expressed in female, whereas prostate stem cell antigen (PSCA) gene was specifically expressed in male (Table 6).

**Table 6:** Differentially expressed genes and specifically expressed genes list in 1-year-old yellow catfish

Sequence ID	FPKM1 (YC1XX)	FPKM2 (YC1XY)	Gene description	Enrichment
comp1014_c0_seq1	2.50E-7	1,331.06	Cytochrome P450 1A (CYP1A)	♀DEG
comp42555_c1_seq1	29,897.90	167.78	Zona pellucida sperm-binding protein 3-like (ZP3)	♀DEG
comp347596_c0_seq1	8,982.63	337.89	Oogenesis-related gene (org)	♀DEG
comp627131_c0_seq1	3,050.23	314.02	Glyceraldehyde 3-phosphate dehydrogenase (GAPDH)	♀DEG
comp157281_c0_seq1	14.52	282.80	Sperm acrosome membrane-associated protein 4-like (SPACA4)	♂DEG
comp368208_c0_seq1	1,383.14	0	Folliculogenesis-specific basic helix-loop-helix (FIGLA)	♀SEG
comp521437_c0_seq1	0	2,120.74	Prostate stem cell antigen precursor-like (PSCA)	♂SEG

*FPKM1* gene expression read value in 1-year-old female yellow catfish, *FPKM2* gene expression read value in 1-year-old male yellow catfish

For 2-year-old yellow catfish, we have found 12 sex differentially expressed genes in male, including CYP1A, 20BHSD, GTHa, zonadhesin (ZAN) (Hunt et al. 2005), growth hormone (GH), cytochrome P450 arom, leptin receptor (LEPR) (Liu et al. 2010; Gong et al. 2013), Sox9a2 (Nakamoto et al. 2009), insulin-like growth factor binding protein 1 (IGFBP1) (Yu et al. 2013), HSP70 (Mayer and Bukau 2005; Tao et al. 2013), RPL15, and myogenin. Only one sex differentially expressed gene was detected in female, which is ppiD (Table 7). GAPDH and GSG1L were found specifically expressed in female, whereas gamete and mating-type-specific protein A (gmsA) and meiosis inhibitor 1 (MEI1) were specifically expressed in male (Table 7).

**Table 7:** Differentially expressed genes and specifically expressed genes list in 2-year-old yellow catfish

Sequence ID	FPKM1	FPKM2	Gene discription	Enrichment
	(YC2XX)	(YC2XY)		
comp19602_c0_seq4	22.61	2.77	Peptidyl-prolyl cis-trans isomerase D (ppiD)	♀DEG
comp3119_c0_seq1	54.36	4,762.43	Cytochrome P450 1A (CYP1A)	♂DEG
comp14556_c0_seq1	96.95	5,826.18	20 beta-hydroxysteroid dehydrogenase (20BHSD)	♂DEG
comp95107_c0_seq1	16.04	769.38	Gonadotropin alpha subunit (GTHa)	♂DEG
comp10436_c0_seq1	170.31	3,218.14	Zonadhesin-like (ZAN)	♂DEG
comp7131_c0_seq1	225.28	4,158.40	Growth hormone (GH)	♂DEG
comp17721_c0_seq1	95.6	1,732.57	Cytochrome P450 arom (CYP)	♂DEG
comp15039_c0_seq1	38.77	664.98	Leptin receptor (LEPR)	♂DEG
comp4302_c0_seq1	72.28	1,034.19	HMG box transcription factor Sox9a2 (Sox9a2)	♂DEG
comp2531_c0_seq1	404.08	5,427.10	Insulin-like growth factor binding protein 1 (IGFBP1)	♂DEG
comp31477_c1_seq3	31.17	362.04	Heat shock protein 70 (HSP70)	♂DEG
comp11313_c0_seq1	100.56	796.7	Ribosomal protein L15 (RPL15)	♂DEG
comp7379_c0_seq1	23.84	161.26	Myogenin	♂DEG
comp666188_c0_seq1	24.17	0	GAPDH protein	♀SEG
comp207731_c0_seq1	13.02	0	Germ cell-specific gene 1-like protein-like (GSG1L)	♀SEG
comp261806_c0_seq1	0	307.89	Gamete and mating-type-specific protein A-like (gmsA)	♂SEG
comp143101_c0_seq1	0	8.42	Meiosis inhibitor protein 1-like (MEI1)	♂SEG

*FPKM1* gene expression read value in 2-year-old female yellow catfish, *FPKM2* gene expression read value in 2-year-old male yellow catfish

### Validation of RNA-Seq Results by qPCR

In order to validate the sexually dimorphic expressed genes identified by RNA-Seq, we randomly selected eight genes from those with differentially expression patterns and from DEGs and SEGs based on their function for qRT-PCR validation, including oogenesis, foliculogenesis, IGFBP1, MEI1, P450, SOX9a2, GTHa, and CYP1A. Comparing the transcriptome data with the qRT-PCR results from seven selected differentially expressed genes, qRT-PCR results were consistent with RNA-Seq results at two developmental stages (Fig. 4). Overall, the qRT-PCR results indicated the reliability and accuracy of the Trinity reference assembly and the RNA-Seq-based transcriptome expression analysis.

## Discussions

The discovered genes provide a baseline for understanding the brain sexual divergence in yellow catfish. In this work, we have sequenced, assembled, and annotated the brain transcriptome of yellow catfish using Illumina HiSeq 2000 sequencing technology. Among those differentially expressed genes, many genes were identified involving sex determination. In addition, a large number of SSRs and SNVs were discovered as genetic markers. We then validated seven selected sexually dimorphic expressed genes by qRT-PCR. The results showed that the sex-related genes were significantly associated with sex determination and sex differentiation indicating the reliability and accuracy of our analysis.

These genes identified are involved in development, metabolism, and biological regulation; some of them have been reported to be associated with sex determination. We identified more XX-related genes in 1-year-old yellow catfish; however, in 2-year-old, most of genes were found to be associated with XY. It is known that the brain of mammals had a remarkable sexual dimorphism in function which is consistent with our study. But the number of identified sex-related genes in the brain was less compared to the gonad, which is consistent with previous studies in other vertebrates (Yang et al. 2006; Mank et al. 2008) and fish species (Manousaki et al. 2014).

The sex-related differentially expressed genes were discussed as follows:

### The Genes Overexpressed in Male Brains

SPACA4 also known as SAMP14, found only expressed in human testis (Shetty et al. 2003), was overexpressed in the 1-year-old male yellow catfish. PSCA is firstly identified as a sex-related gene, specifically expressed in the male brain of 1-year-old yellow catfish in our study.

In 2-year-old yellow catfish, ZAN encodes a sperm protein that is involved in the binding of egg and sperm (Hunt et al. 2005; Hardy and Garbers 1995). LEPR was reported to be highly expressed in testes of zebrafish (Liu et al. 2010). Sox9a2 as a member of Sox9, found overexpressed in male of yellow catfish, has also been reported as a sex-related gene and overexpressed in male rainbow trout (Vizziano et al. 2007) and channel catfish (Sun et al. 2013). IGFBP1 as an insulin-like growth factor binding protein 1, and insulin-like growth factor (IGF), is very important to growth, development, and metabolic regulation in vertebrate. It is reported that IGFBP is critical to gonad development (Yi et al. 2001).

The expression of GH had no difference between male and female in 1-year-old yellow catfish: however, in 2-year-old, the expression in male was found higher than female, which may explain the sex growth dimorphism phenomenon that male grows faster than female in 2-year-old (Liu et al. 2013). RPL15 is firstly identified as a sex-related gene in brain as well as in gonad in yellow catfish, which was previously reported as a molecular marker in teleost phylogenesis (Schulze et al. 2005).

### The Genes Overexpressed in Female Brains

At the developmental stage of 1-year-old, FIGLA, as a folliculogenesis-specific basic helix-loop-helix gene, is identified as a female-related gene. It is considered critical to the development of female germline (Soyal et al. 2000; Pala et al. 2009) and also found to play an important role in XX medaka individuals (Matsuda 2003). ZP3, as a member of zona pellucid (ZP) family, was identified as a female-specific gene in zebrafish (Jiang et al. 2012) and Nile tilapia (Eshel et al. 2014).

In 2-year-old yellow catfish, ppiD identified as DEG and GAPDH and germ cell-specific gene 1 identified as SEGs in female yellow catfish have not been reported as for its role in sex determination.

Compared to our previous study (Lu et al. 2014), CYP1A, GAPDH, GH, HSP70, and RPL15 identified in this study were found as sex-related genes in the brain as well as in the gonad, while other sex-related genes are considered as novel genes firstly found in yellow catfish. In terms of SOX9 family, SOX9a1 and Sox9a2 were identified in the gonad of yellow catfish, whereas only sox92a was found here. IGFBPII were found in the gonad; however, in the brain, IGFBPI were identified. Further investigation is warranted to study the functions of these genes in terms of sex determination.

## Conclusions

This is the first report of yellow catfish brain transcriptome using RNA-Seq technology. In this study, we have assembled and annotated the brain transcriptome and have identified novel sexually dimorphic expressed genes of the yellow catfish. These genes may be candidate genes involved in sex determination. The validation by qRT-PCR indicated the reliability and accuracy of our analysis. In addition, we have also identified many SSRs and SNVs. These genetic markers will assist our understanding of the sex determination mechanism of yellow catfish. Our analysis of sex-related gene expression reveals differences that are likely to be pertinent for the mechanism underlying sex dimorphism.

## Electronic Supplementary Material

Supplementary Table 1 (DOC 92 kb)

Supplementary Table 2 (DOC 212 kb)
