# Supplementary material for: Transcriptomic Analyses Reveal Novel Genes with Sexually Dimorphic Expression in Yellow Catfish (Pelteobagrus fulvidraco) Brain
Source: Mar Biotechnol (NY). 2015 Aug 5;17(5):613–23. doi: 10.1007/s10126-015-9650-z (PMC4540775; doi:10.1007/s10126-015-9650-z)
Supplement: Supplementary file 1 — (DOC 92 kb) [file 10126_2015_9650_MOESM1_ESM.doc]

**Supplementary Table 1. Enriched Gene Ontology (GO) terms of female-biased genes**

| **GO category** | **GO ID** | **GO Term Name** | **P value** |
| --- | --- | --- | --- |
| **Biological Process** | GO:0031333 | negative regulation of protein complex assembly | 0.0069 |
|  | GO:0034587 | piRNA metabolic process | 0.0069 |
|  | GO:0043046 | DNA methylation involved in gamete generation | 0.0069 |
|  | GO:0016337 | cell-cell adhesion | 0.0113 |
|  | GO:0072529 | pyrimidine-containing compound catabolic process | 0.0115 |
|  | GO:0006213 | pyrimidine nucleoside metabolic process | 0.0172 |
|  | GO:0046131 | pyrimidine ribonucleoside metabolic process | 0.0172 |
|  | GO:0006305 | DNA alkylation | 0.0217 |
|  | GO:0006306 | DNA methylation | 0.0217 |
|  | GO:0006304 | DNA modification | 0.0228 |
|  | GO:0044728 | DNA methylation or demethylation | 0.0228 |
|  | GO:0048863 | stem cell differentiation | 0.0228 |
|  | GO:0043254 | regulation of protein complex assembly | 0.0262 |
|  | GO:0090101 | negative regulation of transmembrane receptor protein serine/threonine kinase signaling pathway | 0.0273 |
|  | GO:0007292※ | female gamete generation | 0.0285 |
|  | GO:0048477※ | oogenesis | 0.0285 |
|  | GO:0051129 | negative regulation of cellular component organization | 0.0307 |
|  | GO:0006979 | response to oxidative stress | 0.0318 |
|  | GO:0007126 | meiosis | 0.0341 |
|  | GO:0051321 | meiotic cell cycle | 0.0341 |
|  | GO:0051327 | M phase of meiotic cell cycle | 0.0341 |
|  | GO:0072527 | pyrimidine-containing compound metabolic process | 0.0363 |
|  | GO:0044087 | regulation of cellular component biogenesis | 0.0374 |
|  | GO:0055113 | epiboly involved in gastrulation with mouth forming second | 0.0374 |
|  | GO:0090504 | epiboly | 0.0385 |
|  | GO:0035556 | intracellular signal transduction | 0.0390 |
|  | GO:0009190 | cyclic nucleotide biosynthetic process | 0.0396 |
|  | GO:0009187 | cyclic nucleotide metabolic process | 0.0430 |
|  | GO:0007155 | cell adhesion | 0.0441 |
|  | GO:0022610 | biological adhesion | 0.0441 |
|  | GO:0002011 | morphogenesis of an epithelial sheet | 0.0496 |
| **Cellular Component** | GO:0071944 | cell periphery | 0.0033 |
|  | GO:0043186 | P granule | 0.0079 |
|  | GO:0060293※ | germ plasm | 0.0079 |
|  | GO:0030054 | cell junction | 0.0083 |
|  | GO:0045495 | pole plasm | 0.0094 |
|  | GO:0016323 | basolateral plasma membrane | 0.0123 |
|  | GO:0035770 | ribonucleoprotein granule | 0.0166 |
|  | GO:0048471 | perinuclear region of cytoplasm | 0.0265 |
|  | GO:0005923 | tight junction | 0.0350 |
|  | GO:0070160 | occluding junction | 0.0350 |
|  | GO:0005938 | cell cortex | 0.0357 |
|  | GO:0043296 | apical junction complex | 0.0378 |
|  | GO:0045211 | postsynaptic membrane | 0.0462 |
|  | GO:0097060 | synaptic membrane | 0.0482 |
| **Molecular Function** | GO:0005085 | guanyl-nucleotide exchange factor activity | 0.0053 |
|  | GO:0004016 | adenylate cyclase activity | 0.0117 |
|  | GO:0004601 | peroxidase activity | 0.0181 |
|  | GO:0004889 | acetylcholine-activated cation-selective channel activity | 0.0181 |
|  | GO:0016684 | oxidoreductase activity, acting on peroxide as acceptor | 0.0181 |
|  | GO:0019239 | deaminase activity | 0.0191 |
|  | GO:0009975 | cyclase activity | 0.0202 |
|  | GO:0016849 | phosphorus-oxygen lyase activity | 0.0223 |
|  | GO:0030695 | GTPase regulator activity | 0.0254 |
|  | GO:0016814 | hydrolase activity, acting on carbon-nitrogen (but not peptide) bonds, in cyclic amidines | 0.0254 |
|  | GO:0060589 | nucleoside-triphosphatase regulator activity | 0.0266 |
|  | GO:0016209 | antioxidant activity | 0.0369 |
|  | GO:0005231 | excitatory extracellular ligand-gated ion channel activity | 0.0493 |
