# Supplementary material for: Transcriptomic Analyses Reveal Novel Genes with Sexually Dimorphic Expression in Yellow Catfish (Pelteobagrus fulvidraco) Brain
Source: Mar Biotechnol (NY). 2015 Aug 5;17(5):613–23. doi: 10.1007/s10126-015-9650-z (PMC4540775; doi:10.1007/s10126-015-9650-z)
Supplement: Supplementary file 2 — (DOC 212 kb) [file 10126_2015_9650_MOESM2_ESM.doc]

**Supplementary Table 2. Enriched Gene Ontology (GO) terms of male-biased genes**

| **GO category** | **GO ID** | **GO Term Name** | **P value** |
| --- | --- | --- | --- |
| **Biological Process** | GO:0051258 | protein polymerization | 4.65E-12 |
|  | GO:0043623 | cellular protein complex assembly | 3.41E-10 |
|  | GO:0006184 | GTP catabolic process | 6.42E-09 |
|  | GO:1901069 | guanosine-containing compound catabolic process | 6.72E-09 |
|  | GO:0046039 | GTP metabolic process | 1.01E-08 |
|  | GO:0009143 | nucleoside triphosphate catabolic process | 1.11E-08 |
|  | GO:0009146 | purine nucleoside triphosphate catabolic process | 1.11E-08 |
|  | GO:0009203 | ribonucleoside triphosphate catabolic process | 1.11E-08 |
|  | GO:0009207 | purine ribonucleoside triphosphate catabolic process | 1.11E-08 |
|  | GO:0009154 | purine ribonucleotide catabolic process | 1.15E-08 |
|  | GO:0009261 | ribonucleotide catabolic process | 1.15E-08 |
|  | GO:0006195 | purine nucleotide catabolic process | 1.21E-08 |
|  | GO:0006152 | purine nucleoside catabolic process | 1.31E-08 |
|  | GO:0046130 | purine ribonucleoside catabolic process | 1.31E-08 |
|  | GO:0042454 | ribonucleoside catabolic process | 1.43E-08 |
|  | GO:0072523 | purine-containing compound catabolic process | 1.43E-08 |
|  | GO:1901068 | guanosine-containing compound metabolic process | 1.43E-08 |
|  | GO:0009164 | nucleoside catabolic process | 1.49E-08 |
|  | GO:0009166 | nucleotide catabolic process | 1.56E-08 |
|  | GO:1901292 | nucleoside phosphate catabolic process | 1.63E-08 |
|  | GO:1901658 | glycosyl compound catabolic process | 1.63E-08 |
|  | GO:0034622 | cellular macromolecular complex assembly | 2.84E-08 |
|  | GO:0046434 | organophosphate catabolic process | 2.86E-08 |
|  | GO:0006461 | protein complex assembly | 3.14E-08 |
|  | GO:0070271 | protein complex biogenesis | 3.30E-08 |
|  | GO:1901136 | carbohydrate derivative catabolic process | 3.88E-08 |
|  | GO:0009144 | purine nucleoside triphosphate metabolic process | 6.02E-08 |
|  | GO:0009199 | ribonucleoside triphosphate metabolic process | 6.02E-08 |
|  | GO:0009205 | purine ribonucleoside triphosphate metabolic process | 6.02E-08 |
|  | GO:0009141 | nucleoside triphosphate metabolic process | 6.23E-08 |
|  | GO:0034655 | nucleobase-containing compound catabolic process | 7.95E-08 |
|  | GO:0002504 | antigen processing and presentation of peptide or polysaccharide antigen via MHC class II | 9.61E-08 |
|  | GO:0007017 | microtubule-based process | 1.12E-07 |
|  | GO:0019439 | aromatic compound catabolic process | 1.27E-07 |
|  | GO:0071822 | protein complex subunit organization | 1.33E-07 |
|  | GO:0044270 | cellular nitrogen compound catabolic process | 1.53E-07 |
|  | GO:0042278 | purine nucleoside metabolic process | 1.58E-07 |
|  | GO:0046128 | purine ribonucleoside metabolic process | 1.58E-07 |
|  | GO:0046700 | heterocycle catabolic process | 1.63E-07 |
|  | GO:1901361 | organic cyclic compound catabolic process | 1.79E-07 |
|  | GO:1901565 | organonitrogen compound catabolic process | 1.79E-07 |
|  | GO:0009119 | ribonucleoside metabolic process | 1.97E-07 |
|  | GO:0009150 | purine ribonucleotide metabolic process | 2.65E-07 |
|  | GO:0009116 | nucleoside metabolic process | 2.90E-07 |
|  | GO:0009259 | ribonucleotide metabolic process | 2.90E-07 |
|  | GO:0019693 | ribose phosphate metabolic process | 2.90E-07 |
|  | GO:1901657 | glycosyl compound metabolic process | 3.07E-07 |
|  | GO:0006163 | purine nucleotide metabolic process | 3.35E-07 |
|  | GO:0065003 | macromolecular complex assembly | 4.71E-07 |
|  | GO:0072521 | purine-containing compound metabolic process | 5.80E-07 |
|  | GO:0019882 | antigen processing and presentation | 1.43E-06 |
|  | GO:0043933 | macromolecular complex subunit organization | 1.64E-06 |
|  | GO:0009117 | nucleotide metabolic process | 2.35E-06 |
|  | GO:0044248 | cellular catabolic process | 2.56E-06 |
|  | GO:0006753 | nucleoside phosphate metabolic process | 2.57E-06 |
|  | GO:0055086 | nucleobase-containing small molecule metabolic process | 5.29E-06 |
|  | GO:1901575 | organic substance catabolic process | 8.82E-06 |
|  | GO:0006796 | phosphate-containing compound metabolic process | 1.08E-05 |
|  | GO:0006793 | phosphorus metabolic process | 1.21E-05 |
|  | GO:0009056 | catabolic process | 1.52E-05 |
|  | GO:0022607 | cellular component assembly | 2.17E-05 |
|  | GO:0019637 | organophosphate metabolic process | 2.34E-05 |
|  | GO:1901135 | carbohydrate derivative metabolic process | 3.87E-05 |
|  | GO:0044085 | cellular component biogenesis | 6.69E-05 |
|  | GO:1901564 | organonitrogen compound metabolic process | 0.0002 |
|  | GO:0010035 | response to inorganic substance | 0.0003 |
|  | GO:0006955 | immune response | 0.0004 |
|  | GO:0009408 | response to heat | 0.0005 |
|  | GO:0044281 | small molecule metabolic process | 0.0007 |
|  | GO:0046686 | response to cadmium ion | 0.0009 |
|  | GO:0002376 | immune system process | 0.0014 |
|  | GO:0009266 | response to temperature stimulus | 0.0019 |
|  | GO:0010038 | response to metal ion | 0.0053 |
|  | GO:0044237 | cellular metabolic process | 0.0054 |
|  | GO:0016043 | cellular component organization | 0.0063 |
|  | GO:0071840 | cellular component organization or biogenesis | 0.0096 |
|  | GO:0009628 | response to abiotic stimulus | 0.0116 |
|  | GO:0006814 | sodium ion transport | 0.0145 |
|  | GO:0034765 | regulation of ion transmembrane transport | 0.0145 |
|  | GO:0034762 | regulation of transmembrane transport | 0.0150 |
|  | GO:0042542 | response to hydrogen peroxide | 0.0160 |
|  | GO:0006413 | translational initiation | 0.0165 |
|  | GO:0060027 | convergent extension involved in gastrulation | 0.0181 |
|  | GO:0021534 | cell proliferation in hindbrain | 0.0192 |
|  | GO:0043269 | regulation of ion transport | 0.0208 |
|  | GO:0021592 | fourth ventricle development | 0.0224 |
|  | GO:0044267 | cellular protein metabolic process | 0.0304 |
|  | GO:0000302 | response to reactive oxygen species | 0.0318 |
|  | GO:0008360 | regulation of cell shape | 0.0318 |
|  | GO:0044238 | primary metabolic process | 0.0349 |
|  | GO:0051049 | regulation of transport | 0.0376 |
|  | GO:0021551 | central nervous system morphogenesis | 0.0381 |
|  | GO:0006468 | protein phosphorylation | 0.0386 |
|  | GO:0016310 | phosphorylation | 0.0406 |
|  | GO:0044699 | single-organism process | 0.0422 |
|  | GO:0044710 | single-organism metabolic process | 0.0426 |
|  | GO:0060026 | convergent extension | 0.0428 |
|  | GO:0007623 | circadian rhythm | 0.0474 |
|  | GO:0009410 | response to xenobiotic stimulus | 0.0474 |
| **Cellular components** | GO:0005874 | microtubule | 2.02E-09 |
|  | GO:0043234 | protein complex | 2.19E-09 |
|  | GO:0032991 | macromolecular complex | 1.74E-08 |
|  | GO:0042613 | MHC class II protein complex | 1.77E-07 |
|  | GO:0015630 | microtubule cytoskeleton | 3.24E-07 |
|  | GO:0042611 | MHC protein complex | 1.35E-06 |
|  | GO:0044430 | cytoskeletal part | 2.94E-06 |
|  | GO:0005856 | cytoskeleton | 5.87E-06 |
|  | GO:0005623 | cell | 5.29E-05 |
|  | GO:0044464 | cell part | 5.29E-05 |
|  | GO:0044459 | plasma membrane part | 0.0003 |
|  | GO:0043228 | non-membrane-bounded organelle | 0.0003 |
|  | GO:0043232 | intracellular non-membrane-bounded organelle | 0.0003 |
|  | GO:0001518 | voltage-gated sodium channel complex | 0.0004 |
|  | GO:0034706 | sodium channel complex | 0.0004 |
|  | GO:0005622 | intracellular | 0.0087 |
|  | GO:0043227 | membrane-bounded organelle | 0.0138 |
|  | GO:0043231 | intracellular membrane-bounded organelle | 0.0138 |
|  | GO:0000502 | proteasome complex | 0.0140 |
|  | GO:0034703 | cation channel complex | 0.0151 |
|  | GO:0034702 | ion channel complex | 0.0282 |
|  | GO:0019773 | proteasome core complex, alpha-subunit complex | 0.0349 |
|  | GO:0005852 | eukaryotic translation initiation factor 3 complex | 0.0451 |
| **Molecular Function** | GO:0005200 | structural constituent of cytoskeleton | 6.00E-14 |
|  | GO:0003924 | GTPase activity | 7.64E-09 |
|  | GO:0035639 | purine ribonucleoside triphosphate binding | 4.14E-08 |
|  | GO:0001883 | purine nucleoside binding | 4.23E-08 |
|  | GO:0032550 | purine ribonucleoside binding | 4.23E-08 |
|  | GO:0032549 | ribonucleoside binding | 4.42E-08 |
|  | GO:0001882 | nucleoside binding | 4.52E-08 |
|  | GO:0032555 | purine ribonucleotide binding | 4.52E-08 |
|  | GO:0017076 | purine nucleotide binding | 4.57E-08 |
|  | GO:0032553 | ribonucleotide binding | 5.31E-08 |
|  | GO:0005525 | GTP binding | 1.95E-07 |
|  | GO:0019001 | guanyl nucleotide binding | 2.19E-07 |
|  | GO:0032561 | guanyl ribonucleotide binding | 2.19E-07 |
|  | GO:0043168 | anion binding | 3.56E-07 |
|  | GO:0000166 | nucleotide binding | 3.68E-07 |
|  | GO:1901265 | nucleoside phosphate binding | 3.68E-07 |
|  | GO:0036094 | small molecule binding | 5.72E-07 |
|  | GO:0005198 | structural molecule activity | 2.28E-06 |
|  | GO:1901363 | heterocyclic compound binding | 2.17E-05 |
|  | GO:0097159 | organic cyclic compound binding | 2.29E-05 |
|  | GO:0017111 | nucleoside-triphosphatase activity | 2.75E-05 |
|  | GO:0016462 | pyrophosphatase activity | 3.38E-05 |
|  | GO:0016818 | hydrolase activity, acting on acid anhydrides, in phosphorus-containing anhydrides | 3.58E-05 |
|  | GO:0016817 | hydrolase activity, acting on acid anhydrides | 3.90E-05 |
|  | GO:0043167 | ion binding | 3.92E-05 |
|  | GO:0005488 | binding | 8.65E-05 |
|  | GO:0005248 | voltage-gated sodium channel activity | 0.0006 |
|  | GO:0005272 | sodium channel activity | 0.0015 |
|  | GO:0005524 | ATP binding | 0.0089 |
|  | GO:0032559 | adenyl ribonucleotide binding | 0.0091 |
|  | GO:0030554 | adenyl nucleotide binding | 0.0091 |
|  | GO:0003743 | translation initiation factor activity | 0.0171 |
|  | GO:0003824 | catalytic activity | 0.0223 |
|  | GO:0016787 | hydrolase activity | 0.0300 |
|  | GO:0016772 | transferase activity, transferring phosphorus-containing groups | 0.0315 |
|  | GO:0003913 | DNA photolyase activity | 0.0317 |
|  | GO:0004683 | calmodulin-dependent protein kinase activity | 0.0317 |
|  | GO:0019904 | protein domain specific binding | 0.0355 |
|  | GO:0008135 | translation factor activity, nucleic acid binding | 0.0362 |
|  | GO:0004674 | protein serine/threonine kinase activity | 0.0370 |
|  | GO:0015081 | sodium ion transmembrane transporter activity | 0.0370 |
|  | GO:0022843 | voltage-gated cation channel activity | 0.0378 |
|  | GO:0004672 | protein kinase activity | 0.0401 |
|  | GO:0051015 | actin filament binding | 0.0421 |
|  | GO:0016301 | kinase activity | 0.0496 |
